# Lessons learned from the London Exercise and Pregnant (LEAP) Smokers randomised controlled trial process evaluation: implications for the design of physical activity for smoking cessation interventions during pregnancy

**DOI:** 10.1186/s12889-017-4013-5

**Published:** 2017-01-17

**Authors:** Nikoletta Giatras, Elisabeth Wanninkhof, Miranda Leontowitsch, Beth Lewis, Adrian Taylor, Sue Cooper, Michael Ussher

**Affiliations:** 1 City University London, Cass Business School, 106 Bunhill Row, London, EC1Y 8TZ UK; 2 St George’s University of London, Population Health Research Institute, Cranmer Terrace, London, SW17 0RE UK; 3 Interdisciplinary Ageing Research, Department of Education, Goethe University, Frankfurt am Main, 60323 Germany; 4 School of Kinesiology, University of Minnesota, Minneapolis, MN USA; 5 Plymouth University Peninsula Schools of Medicine and Dentistry, Plymouth, Devon UK; 6 Division of Primary Care, The University of Nottingham, School of Medicine, University Park, Nottingham, NG7 2RD UK

**Keywords:** Process evaluation, Researchers’ perspective, Smoking cessation, Physical activity intervention, Exercise, Pregnant smokers, Qualitative

## Abstract

**Background:**

The challenges of delivering interventions for pregnant smokers have been poorly documented. Also, the process of promoting a physical activity intervention for pregnant smokers has not been previously recorded. This study describes the experiences of researchers conducting a randomised controlled trial of physical activity as an aid to smoking cessation during pregnancy and explores how the effectiveness of future interventions could be improved.

**Methods:**

Two focus groups, with independent facilitators, were conducted with six researchers who had enrolled pregnant smokers in the LEAP trial, provided the interventions, and administered the research measures. Topics included recruitment, retention and how the physical activity intervention for pregnant smokers was delivered and how it was adapted when necessary to suit the women. The focus groups were audio-recorded, transcribed verbatim and subjected to thematic analysis.

**Results:**

Five themes emerged related to barriers or enablers to intervention delivery: (1) nature of the intervention; (2) personal characteristics of trial participants; (3) practical issues; (4) researchers’ engagement with participants; (5) training and support needs. Researchers perceived that participants may have been deterred by the intensive and generic nature of the intervention and the need to simultaneously quit smoking and increase physical activity. Women also appeared hampered by pregnancy ailments, social deprivation, and poor mental health. Researchers observed that their status as health professionals was valued by participants but it was challenging to maintain contact with participants. Training and support needs were identified for dealing with pregnant teenagers, participants’ friends and family, and post-natal return to smoking.

**Conclusions:**

Future exercise interventions for smoking cessation in pregnancy may benefit by increased tailoring of the intervention to the characteristics of the women, including their psychological profile, socio-economic background, pregnancy ailments and exercise preferences. Delivering an effective physical activity intervention for smoking cessation in pregnancy may require more comprehensive training for those delivering the intervention, particularly with regard to dealing with teenage smokers and smokers’ friends and family, as well as for avoiding post-natal return to smoking.

**Trial registration:**

ISRCTN48600346, date of registration: 21/07/2008.

**Electronic supplementary material:**

The online version of this article (doi:10.1186/s12889-017-4013-5) contains supplementary material, which is available to authorized users.

## Background

Smoking cessation during pregnancy is a global public health priority as smoking in pregnancy is the leading preventable cause of morbidity and death among women and infants in industrialised nations [1]. Behavioural support can increase smoking cessation rates in pregnancy by only around 6% and additional aids to cessation are needed [1]. The development of complex smoking cessation interventions during pregnancy have been constrained by limited availability of the process-related data necessary to apply lessons from past studies to improve the design and execution of future studies [1, 2]. In particular, process evaluation provides a means to monitor and document the implementation of interventions in order to understand why an intervention was or was not successful [3]. Besides considering what was delivered, process evaluation helps us to understand how the intervention was delivered. Ultimately, this information can inform researchers, policy makers and practitioners about how the intervention might be implemented in future. We could only identify a few smoking cessation and pregnancy trials which have reported process data and there was very limited information about the experience of those delivering the intervention [4–6].

The purpose of the present study was to conduct a process evaluation of the London Exercise And Pregnant smokers (LEAP) randomised controlled trial, focusing on the experiences of the researchers conducting the trial. The main aim of the LEAP study was to assess a physical activity intervention as an aid to smoking cessation during pregnancy. The trial protocol [7] is published elsewhere and the study methods are summarised here: The 785 women participating in the trial were daily smokers, recruited from antenatal clinics in the South East of England at 10 to 24 weeks of gestation. After enrollment participants were randomised (1:1). Those in the physical activity group (*n* = 392) were offered 14 sessions, combining supervised treadmill walking with physical activity consultations (including exercise diaries and pedometers to encourage non-supervised exercise), plus six sessions of standard behavioural support for smoking cessation. Those in the control group (*n* = 393) received six weekly sessions of behavioural support for smoking cessation alone [8]. The main findings have been published and there was no significant difference in smoking rates between the two study groups at the end of pregnancy [8].

As reported in this paper, we conducted a parallel qualitative-based process evaluation to help understand the challenges associated with trial procedures, and intervention delivery and engagement [9]. We report here the qualitative results of focus groups used to elicit the experiences of researchers who recruited women to the trial, delivered all the interventions and administered the research measures. Further qualitative work, involving interviewing the pregnant women participating in the trial, will be published elsewhere. The aim of this paper was to use the process evaluation data from LEAP to report the broader lessons learned to improve future physical activity and smoking cessation interventions for pregnant smokers.

## Methods

### Design

A qualitative methodology was chosen for this process evaluation of an RCT as it would allow an in-depth and non-hypothesis approach to investigating the experiences of the researchers involved in the trial. We adopted a qualitative descriptive approach, which is suitable for gathering professionals’ experiences and involves a rich description of experiences and events [10]. Focus groups were chosen as they allow the study of a group perspective among a particular set of people who share a set of common experiences, especially where the subject of study is little understood and talked about infrequently in day-to-day life or professional practice. It is also a strong method in allowing topics to come to the foreground that might not be talked about in one-to-one settings such as qualitative interviews. As a form of group interview, participants are encouraged to talk to one another: asking questions, exchanging anecdotes and commenting on each other’s experiences and points of view [11]. Moreover, focus groups are considered particularly valuable when developing and refining health education messages and interventions [8].

### Focus group participants

Following an invitation from the principal investigator (MU), all six researchers employed on the trial volunteered to participate in both focus groups. The six researchers comprised three midwives (researchers 1, 2 & 6 as labelled in results below) and one nurse (researcher 3) all with extensive experience of providing behavioural support for smoking cessation in pregnancy, plus one nurse (researcher 4) and psychologist (researcher 5) without previous experience of providing this support. The researchers had been recruited to work on the trial through an advert in a national newspaper and on university websites.

Only one of the researchers had experience of recruiting to trials (R3) and none of the researchers had experience of promoting physical activity. All the researchers were trained to NHS Centre for Smoking Cessation and Training standards during a two-day course [12], plus two days training in physical activity promotion during pregnancy. Two further researchers who had worked on the trial were approached to participate but were not available.

### Ethics and consent

The trial was approved by Wandsworth Research Ethics Committee and trial participants gave written informed consent. For the present study, all six focus group participants were informed about taking part in the evaluation and gave written informed consent.

### Topic guide and procedure

Consistent with MRC recommendations for process evaluation, the qualitative data was collected and analysed iteratively so that themes that emerge in early interviews can be explored in later ones [9]. Two focus groups were conducted, with the same six researchers, to explore their experience of delivering the intervention. The first focus group was conducted during trial recruitment (June 2012), so that the researchers could report on issues while the trial was active. The second focus group was conducted during the trial follow-up period (January 2013). A topic guide for the first focus group, with open-ended questions, was developed for understanding the researchers’ perspectives on the trial (see Additional file 1). It focused on recruitment and retention factors (such as timing, methods for contacting potential participants), participant-related factors (such as health problems, social networks); the nature of the RCT (randomisation, use of technology, encourage exercise intervention) and the interaction between researchers and participants.

The first focus group lasted 60 min, was audio-recorded and transcribed verbatim. Topics discussed included recruitment, retention, exercise intervention and general trial implementation. The discussion was facilitated by a qualitative investigator (ML) not known to the focus group participants and who was not involved with the main trial. In addition, a co-facilitator, who did not take part in the discussion, took field notes of the focus group to provide background information for the transcription and analysis. After an initial analysis of the transcript and notes by three of the authors (NG, EW and MU) several areas were identified for further exploration in the second focus group, these included: exercise (suitability of treadmill exercise for pregnant women, alternate types of exercise such as yoga, Pilates, swimming or group exercises); communication with participants, suitability of the environment (hospital, children’s centre, sports centre) and psycho-social aspects of the participants lives which may have affected attendance to sessions or retention [13, 14].

The second focus group was also facilitated by a qualitative investigator (NG), again, not known to the participants and not involved in the main trial, with a co-facilitator taking notes. The topic guide for the second focus group is presented as Additional file 1. This focus group lasted 90 min, was recorded and transcribed verbatim.

### Analysis

The analysis was guided by the framework described by Braun and Clarke [15]. Initial coding was undertaken independently by three investigators: EW (researcher on trial and member of focus groups), NG (lead second focus group) and MU (chief investigator for the trial), by reading and familiarising themselves with the transcripts and developing initial codes from the data. Following this the team assigned codes to lines, sentences and phrases in the transcripts, similar codes were ordered into sub-themes, and then codes and sub-themes ordered to create themes. Themes were then reviewed and refined through discussions (EW, NG, MU) to ensure that they accurately reflect the data and what was said by the participant. This process of investigator triangulation allowed for internal validity [16].

To facilitate data management and analysis, NG used the qualitative software NVivo 9 (NVivo qualitative data analysis software; QSR International Pty Ltd Version 9, 2010). Themes are illustrated by selected anonymised quotes which are characteristic of the data. Reporting of the qualitative data is consistent with RATS (Relevance, Appropriateness, Transparency, Soundness) guidance [17].

## Results

We considered that data saturation was achieved and identified five themes related to the difficulties and resources faced by the researchers delivering a physical activity intervention to pregnant smokers. They were: (1) nature of the intervention, (2) personal characteristics of trial participants, (3) practical issues, (4) researchers’ engagement with trial participants and (5) training and support needs. In the following report, FG = Focus Group and R refers to the number of the researcher (i.e., 1 to 6 as indicated above).

### Nature of the intervention

#### Intensive nature of physical activity intervention

Participants attended a median of 4 out of 14 treatment sessions in the intervention group and 3 out of 6 sessions in the control group [8]. Researchers expressed concern that the supervised treadmill exercise was a significant commitment for trial participants that are pregnant. For example, *“Two sessions per week for an exercise group… it was very off-putting…”* (R1, FG1). They felt that some trial participants were put off by the treadmill exercise. “*One trial participant said, “It’s not working for me, going on the treadmill, let’s just chat”.* (R2, FG1). The researchers were cautious about encouraging the women to exercise at higher intensities: *“Some people would go pretty slowly… you’d try to get them to speed up a bit sometimes”* (R2, FG1) *“I was keen not to overly push them and then put them off.”* (R4, FG1).

#### Lack of tailoring

Researchers advised that the intervention could have been more tailored to women’s preferences (e.g., number of intervention sessions, exercise mode):*“There isn’t a ‘one plan fits all’, everyone’s different.”* (R4, FG1). There was also a concern that there were few opportunities for community-based exercise tailored to pregnancy, beyond the main trial intervention: *“I thought there might be people there [sports centre], personal trainers, who would have had pregnancy training, and there wasn’t anybody”* (R2, FG2).

#### Simultaneous behaviour change: stopping smoking and increasing physical activity

Helping the trial participants to change two health behaviours (stopping smoking and increasing physical activity) simultaneously was seen as challenging: *“Maybe two things was a bit too much. Maybe we should get them to stop smoking or to be fit and then, tackle the other bit.”* (R2, FG2). Conversely, exercise was viewed as a helpful distraction: *“If you’re giving up smoking, all you think about is a cigarette. If you’re trying to get fitter and do exercise, by not just focusing on the cigarettes… sometimes it makes it easier.”* (R4, FG2).

### Personal characteristics of trial participants

#### Socio-economic background

Trial participants frequently came from deprived backgrounds (including a mixture of factors such as low income, single parenthood, poor housing, unstable partnerships, drug/alcohol abuse, unemployment), which meant that their health was not high on their agenda [18]. This may have made demands that competed with participation in the trial: *“Their lives are so chaotic… they find the whole process keeping to appointments difficult.”* (R2, FG1). *“There were a few where their partner was an alcoholic”* (R5, FG2) *“… they had so many other problems apart from smoking…”* (R3, FG2).

Financial need was also seen as a barrier to attendance: *“They have to have that money in the first place [to travel to appointments] and it’s quite expensive, the bus”* (R3, FG2). The cost of exercising, beyond the supervised exercise provided by the study, was also identified as a problem: *“They would often say, ‘Well, I can’t afford to go swimming’. A lot of them were on income support, and it surprised me why there weren’t more initiatives to encourage low incomes into gyms.”* (R4, FG2).

#### Psychological issues

Researchers reported that many trial participants had an external locus of control and low self-efficacy and this may have affected their confidence for quitting smoking and for attending treatment sessions: *“They haven’t done very well at school, they’ve gone through bad relationships, and everything is sort of out of their control.”* (R4, FG1). *“People have never said very positive things to them… people have been telling them what they’ve been doing wrong.”* (R4, FG2).

Researchers also reported that it was evident from their conversations with women, that some women were depressed but that they often accepted being ‘down’ as a routine part of life. Researchers found it challenging to know how to address this as it was beyond the focus of the intervention: *“I got very worked up about it [high score on depression questionnaire]. I thought, oh my God, these women are ready to jump off bridges! You don’t know if that’s going to deter them from coming back, if you start talking about their emotional stability.” (R2, FG2)… “There’s always the issue, ‘Oh, you’re going to get Social Services involved and my baby is going to get taken away!’” (R4, FG2)* This issue not only affected participants’ capacity to work through the trial, but also affected the researchers’ role of retaining participants at the same time acknowledging their challenging mental health status.

#### Pregnancy trajectories

For adherence, the stage of pregnancy was considered important: *“They tend to be most enthusiastic at the beginning of a pregnancy, but they were usually very often a bit sick [at] 14 weeks, they were usually over the sickness, they weren’t that big… by the time they got to 30–31 weeks, they weren’t keen on doing the exercise (murmurs of agreement)… just too heavy.”* (R2, FG2).

Regarding encouraging the women to adhere to the exercise prescription, the researchers were cautious when women reported any pregnancy related symptoms. For example, early on in the pregnancy “women felt nauseous”; as the pregnancy progressed they found it “painful to walk”, “had anaemia”, and were “limited by backache and pelvic girdle pains”. In more severe cases some women withdrew from the interventions: *“I had a woman who was diagnosed with placenta-previa; as soon as she found out she stopped coming back… she’s not taking any risk.”* (R1, FG2)

### Practical issues

#### Randomization

Trial randomisation took place in a hospital, a community-based children’s centre, or a leisure centre. Researchers used an internet-based system to randomise the women and to complete study forms on-line. This system was often a source of frustration for the researchers as it required a reliable internet connection: *R5: “Even with the good connections, the patient would fill the page up, try and go onto the next page and then, “Oh, what’s happened?” “Oh, it’s all gone blank; OK, we’ll have to start again!” (murmurs of agreement) R3: “Oh, that was so irritating” R5: “Yeah, that was quite embarrassing, because you’re just trying to go with the flow of the session, (murmurs of agreement)” R3: “Then if there’s no signal from this room, you have to move to another one.” (FG2)*

The technical difficulties could affect the important rapport researchers were trying to establish with the trial participants of whom some were particularly vulnerable and therefore more prone to leaving the trial early. Finding an appropriate room, whether for Internet or physical exercise, was often a challenge.

#### Room allocation

The researchers consistently found it challenging to find a dedicated treatment room: *“We had a room set and organised and just as I started, the hospital said: ‘No, you can’t have that room.’”* (R4, FG1) *“… getting tossed out of my room and having patients arrive and I had nowhere to see them, it was hard.” *(R3, FG1).

#### Child care

Many women were accompanied by children. If available, a crèche (i.e., child care facility) was offered and paid for. Researchers felt that the women valued this but it was considered costly: *“We could book child care, but that was always very expensive and then they wouldn’t turn up and you’d still have to pay.”* (R2, FG2).

#### Financial incentives

Paying the women £7 travel expenses at each visit was viewed as a vital incentive:

*“A lot of them would tell me what they’d use their £7 for. Some would say: ‘This will buy me dinner’… If you come twice a week and they do the follow ups, they have £120”* (R2, FG1). This underscores the previously mentioned difficult socio-economic situations of many of the trial participants.

#### Location of treatment room

The interventions were delivered in a hospital, a community-based children’s centre, or a leisure centre. A hospital location was seen as ideal as it was an easy to find landmark with bus routes stopping at the door and it was felt that women were reassured by the clinical setting: *“They like to come to the hospital…”* (R6, FG1) *“They can find the hospital.”* (R2, FG1). The same did not apply to children’s or leisure centres. *“I tried to get people to come to the children’s centre, but they kept getting lost or just didn’t turn up.” *(R3, FG1). *“A lot of women will have never had anything to do with children’s centres, so they wouldn’t even know what children’s centres are.” (*R6, FG1).

#### Monitoring physical activity

The trial participants assigned to the intervention arm of the RCT were equipped with pedometers and asked to keep an exercise diary, which the researchers considered beneficial: *“My participants were quite keen to tell me if they had been for a walk, or how many minutes they have been walking for.”* (R5, FG2).

There was consensus that the pedometers worked as well: *“People would very often do a bit more walking, because they wanted to see the step counter to go up.”* (R2, FG1). *“When they look at the pedometer, and they’ve done about 1800 [steps] for a whole day, then they realise I am not as active as I thought I was.”* (R1, F2G).

### Researchers’ engagement with trial participants

#### A friendly and professional person to talk to

The face-to-face contact with the researcher was seen as the most valued element of the intervention: *“For most of them it was the contact… I don’t think that many people give them much time … somebody sitting there with them who was prepared to listen.”* (R4, FG1).

Researchers also recognised the benefit of their professional role as a nurse or a midwife: *“It’s certainly helped me in engaging with them and they like the fact that they feel they’re talking to somebody who has got some medical training”* (R4, FG2). Again, this had a double effect: trial participants positively acknowledged the researcher’s professional background, and this background helped the researchers communicate with the trial participants in a patient-centred way, often going beyond their call of duty: *“I would actually make sure that people knew I was a midwife. I would go and find their blood results, and anything I could really do to keep them interested.”* (R2, FG2).

#### Non-judgemental nature of researchers

Researchers were aware of the importance of being non-judgemental. *“Some of them are very worried about failing and I just make sure that they know that I’m not going to be criticising them and that we’ll try this, if it doesn’t work we’ll try something else. I’ve tried to make them feel I’m not there judging them.”* (R2, FG1).

#### Communication

Smoking status for all pregnant women was recorded in the hospital at the first antenatal-booking visit and the midwife informed pregnant smokers that they would be telephoned to be offered smoking-cessation support. The researchers were required to call the women and to offer a place on the trial or offer usual NHS cessation-support. However, they observed that “phoning and leaving verbal messages” was not as effective as texting, either during recruitment or during the ongoing trial: *“The texts were much better… they’re used to that form of communication, but also because they could think about it.”* (R3, FG2).

#### Deviating from the intervention protocol

In order to fit the exercise and counselling within the allocated time frame of about an hour, researchers often slightly adapted the trial protocol by providing some of the counselling while the woman was on the treadmill. This seemed beneficial as it kept the women going and made the session slightly more informal and friendly: “*Inevitably, when they were on the treadmill a little bit of counselling would go on.”* (R4, FG1).

Occasionally a participant was unable to attend the session due to work commitments, so the researcher adapted the session by accompanying the participant on a walk outside: *“I met one woman in her lunch hour and I would walk her around the block a few times.”* (R2, FG2). *“This girl was a waitress in a pub, she worked such long hours I could never get her away, so I’d go and meet her out in the car park [to go for a walk].”* (R3, FG2).

### Training and support needs

Researchers noted several areas in which they could have benefitted from extra training and support.

#### Teenagers

Researchers identified teenage smokers (11% of study participants) as a distinct group. They reported that they may have benefited from training specifically for younger smokers: *“I don’t know if it was just kind of me, but I just sort of felt there was no connection, so I never really got on that well with teenagers. I thought there must be something I can do differently, but I never really discovered what that was. But I think there’s probably strategies or techniques that work better for younger girls.”* (R2, FG2).

Researcher said that teenagers often came with a friend and they found it difficult to manage these interactions: *“…which sometimes would mean they’d then get a bit giggly and a lot of “in” jokes I probably found it easier if they came on their own, but they probably wouldn’t have arrived, they needed support.*” (R2, FG2).

#### Deprivation

As reported in the section above on ‘Socio-economic background’, the researchers often had to deal with women with complex issues related to deprivation and they expressed the need for further training and support for knowing how to deal with these issues, especially when they impacted on smoking cessation.

#### Partners and friends

Partners and friends who smoked were considered a major barrier to smoking cessation. However, researchers had nothing at hand to deal with this set-up: *“Getting the other person to stop smoking never seemed to work, definitely not with the exercise intervention, because they couldn’t really use the treadmill.”* (R2, FG2).

#### Stage of pregnancy

As presented in the section above on ‘Pregnancy trajectories’ the women were seen to have different needs at different stages of pregnancy and researchers requested more training on this topic.

#### Post-partum return to smoking

Researchers provided brief advice about preventing post-partum return to smoking but they expressed the need for further training in offering more comprehensive support in the follow-up assessment sessions after the baby was born: *“Support is needed maybe a month after the baby is born, although it’s difficult…” (*R4, FG2). There were suggestions for what might motivate women to avoid returning to smoking: *“It’s little things…they don’t want the smell on the baby’s clothes. I don’t want my baby to be in a smoking environment.”* (R4, FG2).

## Discussion

To the best of our knowledge, this is the first study to explore perceptions of researchers’ delivering a smoking cessation and physical activity intervention to pregnant smokers. This study provides novel insights into researchers’ perspectives of the intervention and highlights several aspects of the intervention and trial design that may affect recruitment, retention and adherence. The information elicited from the focus groups has many implications for the design of a smoking cessation trial incorporating physical activity for pregnant smokers. It was evident that the trial and its researchers made great efforts to accommodate the needs of the women and that these were generally positively received, including child care provision, re-imbursement of travel expenses, having healthcare professionals deliver the intervention, the choice of venue for appointments and using text messaging as the predominant mode of communication.

### Simultaneous behaviour change

Researchers were ambivalent as to whether exercise should be promoted simultaneously as smoking cessation. Similarly, the literature cites wide variation in the timing of exercise programmes for smoking cessation, at least for smokers in general, [19]. In some instances, it has been recommended that exercise and smoking are changed sequentially, rather than simultaneously [20–23]. Of relevance here, there is evidence that smokers who have achieved abstinence have higher confidence for increasing exercise than those only planning or preparing to quit [21]; which supports the notion of increasing exercise when abstinent. However, this might limit the potential for exercise alleviating withdrawal symptoms and cravings in the critical first days and hours of a quit attempt [24]. Hence, others support the notion that a physical activity intervention should commence several weeks before quitting, enabling the smokers to cope with increasing their activity before starting to quit smoking [25]. Finally, there is also some indication of quit rates being higher when exercise is increased at the same time as quitting smoking, as opposed to doing this sequentially [23].

In regard to pregnant smokers, it may be unrealistic and unethical to expect women to delay quitting while they are taking part in a preparatory exercise programme as they are generally concerned about harm to the foetus during continued smoking; also, health professionals would most likely encourage them to quit as early as possible in their pregnancy, unless there is good evidence that delaying quitting will increase their chances of succeeding. Equally, if the exercise programme is delayed until further into the quit attempt, the woman will lose the potential benefit of exercise for ameliorating the withdrawal symptoms, including strong cravings, which are prominent in the first weeks of abstinence [26]. Therefore, as is common with smokers in general [19], and even more so for pregnant smokers, the most beneficial and practical option may be to combine exercise and smoking cessation early in the quitting process.

### Deprivation and mental health

In the main report of the trial we observed that 18% of women were classed as depressed [8], a prevalence which is slightly higher than that reported for pregnant women in general [27]. This is not surprising, as women who are depressed have an increased likelihood of smoking during pregnancy [28]; while, women with mental disorders during pregnancy may have high motivation to quit, but find it extremely challenging to quit [29]. Also, there is little data on the effects of smoking cessation interventions during pregnancy among women with poor mental health, as these women are often excluded [28]. Furthermore, around three quarters of the women in the present study had low scores for self-efficacy for quitting smoking [8]. Perhaps this was a reflection of the difficult and deprived lives they had, and may have contributed to preventing them from succeeding at various things throughout their lives. Public health and medical sociology studies have long pointed to the connection between low socio-economic status and poor health outcomes, as well as motivation to invest in health or even seek medical advice [30].

### Text messaging

Our observations are consistent with a recent feasibility study showing that text messages have the potential to be used for delivering comprehensive and tailored self-help support for smoking cessation during pregnancy [31], although it is not clear whether text-based support is acceptable and useful for promoting physical activity during pregnancy.

### Post-partum return to smoking: Partner and peer support

The researchers expressed the need for more support for preventing postpartum return to smoking. It is well known that a substantial proportion of women who quit smoking during pregnancy will return to smoking in postpartum [32]. Few smoking cessation trials provide and document support for preventing postpartum return to smoking [33] and extension of the period of support for women to stop smoking into postpartum has been proposed [34]. There are various reasons for postpartum return to smoking, including having a partner who smokes [35]. A review concluded that eliciting peer and partner support is positive and can support women to stop smoking, but is a challenge to put into practice [1].

Many pregnant smokers suspend their smoking for the duration of pregnancy or they commit to ‘temporary abstinence’ for pregnancy [35–37]. Whilst, younger women are generally unaware of the specific impact of smoking on their developing baby [38]. This demonstrates the need to target information specifically to different groups of women depending on age, social support and decision-making processes particularly in the time after they have given birth. Several small studies of peer support for smoking cessation in pregnancy have produced promising results [39–41], especially in teenagers, and larger more rigorous studies need to be conducted.

### Lessons learned: a summary of what worked well

Text messaging proved an effective form of communicating with the women. Texts were used for recruitment, for confirming appointments, and for maintaining contact between appointments. Texts were regarded as highly convenient and economical and were well received by the participants (see Table 1).

**Table 1:** Lessons learnt and implications for future interventions: physical activity for smoking cessation during pregnancy

Lesson learnt	Evidence from LEAP trial	Implication for future research and practice
1) Text messaging	Effective form of communicating, convenient and cost-effective.	Include text messaging as a means of communicating with participants.
2) Pedometers	Women enjoyed wearing them particularly because they could see the immediate results.	Use ‘gadgets’ that are simple to use yet effective and empowering for participants.
3) Financial incentives	Facilitated intervention attendance particularly amongst women from deprived backgrounds.	Identify a way of easing financial burden (i.e. travel costs) on women to enable them to attend sessions. Conduct further research on financial incentives for smoking cessation.
4) Intensity of exercise	Researchers were hesitant to encourage women to exercise at higher intensities and not certain how to adapt exercise for different stages of pregnancy.	Current guidelines suggest there is no need to adapt exercise for pregnant women with no medical or obstetric complications; consider revising guidelines.
5) Frequency of visits is too high	Face-to-face support may need to be combined with self-help strategies.	Research is needed to determine how effective this self-help support is for promoting exercise in pregnancy.
6) Training needs	Targeted training would have been beneficial.	Include more and specific information on (i) how to talk to teenagers about smoking, (ii) how to deal with deprivation related issues drug/alcohol abuse, (iii) family/friend/partner presence in session, (iv) stage of pregnancy, (v) preventing postpartum return to smoking.

Pedometers, worn by participants in the physical activity arm of this trial, received positive reviews. Women enjoyed wearing the pedometers, particularly because they could see the immediate results of their efforts (see Table 1). At each support session, participating women were encouraged to increase their step count by about 10%. This proved beneficial as many women either met or exceeded their step-goals. These findings are consistent with findings from a recent systematic review in the general population [42] which showed that pedometer users increased their physical activity by almost 2500 steps per day more than control participants. Pedometers have been shown to increase activity levels in women [43] and have been shown to be acceptable during pregnancy [44] and among pregnant smokers [45] .

The financial incentives (i.e., £7 travel payment for each session attended) seemed to facilitate intervention attendance, particularly amongst the women from deprived backgrounds, which seemed to mainly be attending for this reason (see Table 1). This finding is consistent with reviews of financial incentives in pregnancy [46] and with the findings of a recent UK-based trial [47], in which women receive incentives on confirmation of successful abstinence from smoking.

To aid accessibility, three different treatment venues were offered, including being offered children’s centres based in deprived areas. Participants generally preferred to attend in hospitals. An intervention may have different effects in different contexts, even if its implementation is the same or very similar across contexts [48], although in this case our quantitative work did not show any difference in smoking abstinence according to the context (i.e., venue) in which the intervention was delivered. We can only consider why the women preferred attending at the hospital and this appeared to be because of the central location and a preference for a clinical environment. Hospitals are more often associated with antenatal care and in a hospital setting participants may find it easier to make the link between stopping smoking and their unborn baby’s health. It is not clear what other steps could be taken to encourage women from deprived backgrounds to attend appointments. Pregnant smokers generally prefer one-to-one to group support [49] and offering home visits may have increased attendance, but it was impractical and costly to offer this on an extensive basis.

Researchers presenting themselves to the women more as health professionals than as exercise specialists appeared to facilitate the women’s engagement with the exercise programme. Unlike the previous points, this approach had not been foreseen in the trial procedure, but was recognised by the researchers whilst undertaking the trial.

### Lessons learned: summary of what worked less well

Researchers were hesitant to encourage women to exercise at higher intensities and were often at a loss for how to adapt the exercise to the different stages of pregnancy and to pregnancy related symptoms, such as nausea, backache and fatigue (see Table 1). Intensity has been cited as the most difficult component of an exercise regimen to prescribe for pregnant women with current guidelines suggesting that there is no need to adapt exercise for pregnant women with no medical or obstetric complications [50]. Many women in this trial did not experience complications during the trial, however they had gained weight and their bodies were changing on a weekly basis. Therefore, it may be unrealistic to expect pregnant women to follow the same exercise guidelines as non-pregnant women.

The researchers felt that the frequency of visits (14 sessions for the intervention group vs 6 sessions for the control group) was too high, and that face-to-face support may need to be combined with self-help strategies (e.g., self-help manuals and digital support such as text messaging, phone apps and websites) (see Table 1). There is evidence that self-help interventions are effective for smoking cessation during pregnancy [51] and text message support may be an appropriate intervention in this context (see comments above).

Researchers agreed that more training targeted specifically to teenagers and women from deprived backgrounds would have been beneficial (see Table 1). Specifically, researchers would have found it useful to have more and specific information on the following: (i) how to talk to teenagers about smoking (a study by Hill and colleagues [38]) with young pregnant smokers has highlighted similar findings, with midwives agreeing that having resources relevant to teens would make talking to them easier); (ii) how to deal with complex issues related to deprivation (e.g., drug/alcohol abuse, poor housing) which have an impact on smoking cessation, (iii) how to manage sessions when a friend, partner or other family member, was present (iv) Those delivering a physical activity intervention may also benefit from training to be more confident about what is reasonable to expect of women at different stages of pregnancy or with different complications. Further research is needed on this topic, while noting that each pregnancy is different and one approach will not suit all women. Interventions, as well as expectations, may need to be tailored for individual women.

#### Strengths and limitations

The major strength of this paper is the provision of novel and detailed information from researchers on how to effectively deliver and adapt an exercise intervention for pregnant smokers. The results are strengthened by the provision of the researchers’ views on two separate occasions seven months apart. All researchers’ in the first focus group also participated in the second focus group. Conducting two focus groups allowed further exploration of issues identified in the first focus group; this also generated richer data. Furthermore, those taking part in the focus groups could be considered as experts; all the researchers had substantial experience in implementing the trial and delivering the intervention as they had been in post for approximately three years at the time of the first focus group. One of the researchers contributed to the analysis and it was felt that this “insider view” aided the interpretation of the findings, which is in line with participatory research [52].

There is limited data on how, in practice, smoking cessation specialists promote changes in multiple health behaviours and, specifically, how physical activity is encouraged [23]. This study sheds some light on this, albeit with the focus being on pregnant smokers. It is important to acknowledge that this intervention, and associated process evaluation data, were focussed on pregnant women in the UK and in London specifically; however, the women in the trial were generally representative of women who smoke [8]. The findings of this qualitative evaluation could be transferable to primary and secondary care settings in the UK, although the findings may be less relevant to smokers outside the UK.

## Conclusion

These findings add to the limited process data available concerning the challenges of implementing smoking cessation interventions for pregnant women. The results recognise that we are dealing with a particularly vulnerable group of women from deprived backgrounds. In this context, future studies can learn from the data presented here, combined with qualitative data in which the women are encouraged to share their experience of the trial and interventions (to be published elsewhere), in considering how interventions can be further tailored to the women’s needs. In addition, future studies need to consider providing more comprehensive training for those delivering the intervention, both to deal with deprivation related issues as well as dealing with other under researched challenges such as catering for teenage smokers, managing smokers’ friends and family who attend cessation sessions, and offering advice about avoiding post-natal return to smoking.

## Additional file

Additional file 1: Topic guides for focus group 1 and focus group 2. (DOC 30 kb)

## Abbreviations

AT: Adrian Taylor
BL: Beth Lewis
EW: Elisabeth Wanninkhof
FG1: Focus group 1
FG2: Focus group 2
LEAP: London Exercise And Pregnant smokers randomised controlled trial
ML: Miranda Leontowitsch
MU: Michael Ussher
NK: Nikoletta Giatras
R1: Researcher 1
R2: Researcher 2
R3: Researcher
R4: Researcher 4
R5: Researcher 5
R6: Researcher 6
SC: Sue Cooper
