# Supplementary material for: Lessons learned from the London Exercise and Pregnant (LEAP) Smokers randomised controlled trial process evaluation: implications for the design of physical activity for smoking cessation interventions during pregnancy
Source: BMC Public Health. 2017 Jan 17;17:85. doi: 10.1186/s12889-017-4013-5 (PMC5240296; doi:10.1186/s12889-017-4013-5)
Supplement: Additional file 1: — Topic guides for focus group 1 and focus group 2. (DOC 30 kb) [file 12889_2017_4013_MOESM1_ESM.doc]

**Focus Group 1: Topic Guide**

**Recruitment and retention**

- Tell me about the recruitment of women to the trial.
- Tell me about retaining women in the trial.
- Tell be about your views on the use of incentives (*e.g., travel payments to participants*)
- How did women react to being randomized (exercise or control group)?

**Views on exercise intervention**

- Your role in implementing the intervention:
  - What were your expectations of the intervention?
  - How did you deal with the detailed paper work/protocols?
  - How quickly did you become confident with all the aspects that needed covering in each of the sessions?
  - How did you balance smoking cessation and physical activity in the same session?
  - What would you have done differently, if it had been your trial?
- Your role in supporting pregnant women to stop smoking:
  - Training prior to trial
  - Experience of working with women during the trial
  - Most valuable knowledge acquired
  - The value of the different components:
    - Exercise consultation / counselling
    - Supervised exercise
    - Pedometers
    - Exercise diaries
  - Why do you think some women faired better than others?
- Women’s perspective:
  - What did they value most?
  - Did the intervention help them quit?
  - When were the women most animated?

**The trial in general**

- What has been particularly challenging?
- What has been particularly rewarding?
- What would you have done differently?
- How has your professional background shaped your researcher role? E.g.
  - Recruiting women
  - Delivering the intervention
  - Interacting with participants
  - Liaising with other health professionals

**Focus Group 2: Topic Guide**

How were sessions affected when participants were accompanied by others (e.g. partner, family member, friend, own children......)? If you adapted the session to accommodate the other person, did it matter whether the participant was in the exercise or control group?

Did you ever need to change aspects of the sessions to take particular characteristics of participants into account (e.g., ethnic origin, teenagers, dyslexia, mental health issues.....)? Were certain ladies more suited to one arm of the study than the other?

From your point of view, which of the many incentives offered were most successful in getting women to engage with the programme? Which incentives appeared to make little difference?

- paying travel expenses
- paying for childcare
- baby grows
- £10 store vouchers for completed follow-up questionnaires
- £5 store vouchers sent with follow-up questionnaires to encourage women to send them back
- prize draw
- refreshments
- pedometers (exercise group)
- exercise booklet/exercise DVD (exercise group)
- Televisions (exercise group)

Are there any other incentives or strategies which you have found helpful when working with participants (e.g. choice of venue for visits, particular leaflets/posters/props, use of technology such as text messaging......)

Can you give examples of any instances when you needed to deviate from the LEAP Study protocol?

In your opinion, what personal characteristics are important when working as a smoking cessation advisor for pregnant smokers? Do you think the professional background of the advisor make a difference?
